# *Rango Cards*, a digital game designed to promote a healthy diet: a randomized study protocol

**DOI:** 10.1186/s12889-018-5848-0

**Published:** 2018-07-24

**Authors:** Carolina Martins dos Santos Chagas, Tiago Barros Pontes e Silva, Luiggi Monteiro Reffatti, Raquel Braz Assunção Botelho, Natacha Toral

**Affiliations:** 1 University of Brasilia School of Health Sciences, Darcy Ribeiro College Campus, Brasilia, Federal District, Postal Code 70910-900 Brazil; 2 University of Brasilia Institute of Arts, Darcy Ribeiro College Campus, Brasilia, Federal District, Postal Code 70910-900 Brazil; 3 Fira Soft, SGAS 904 Complex A, ASCEB, Block J, 2nd Floor, Brasília, Federal District, Postal Code 70390-040 Brazil; 4 University of Brasilia School of Health Sciences, Graduate Program in Human Nutrition, Darcy Ribeiro College Campus, Brasilia, Federal District, Postal Code 70910-900 Brazil

**Keywords:** Digital game, Healthy diet, Food and nutrition education

## Abstract

**Background:**

Several food and nutrition education actions have been described in the literature, with emphasis on the recommended use of innovative methods when addressing a young audience. Digital games are an attractive, dynamic, and motivating resource for teaching and learning practices, and adolescents form the group that readily accepts and adopts new technologies. Adapting dietary and nutritional guidelines to change dietary behavior is a challenge, and game-based learning has several benefits that can be used in this sense. Thus, this study aims to outline a nutritional intervention for school-aged adolescents from the Federal District, Brazil, whose object is a digital card game aimed at promoting healthy dietary practices.

**Methods:**

In this randomized study with intervention and control groups, we propose a nutritional intervention for adolescents studying in Federal District private schools. The intervention group will be introduced to *Rango Cards*, a digital game specifically developed for this study. The purpose of the game is to present the concept of an adequate and healthy diet using simple information in a playful context. This game features cards for foods/meals, characters, and healthy habits. The players’ choices may lead them to winning or losing. Theme selection and phase order were designed to provide a learning experience. The control group will not receive any material during the study. Both groups will complete questionnaires before and after the intervention. The game is expected to improve food knowledge and self-efficacy in the adoption of healthy practices, thus contributing to appropriate dietary consumption.

**Discussion:**

The game was designed as a food and nutrition education tool based on Brazilian dietary guidelines. We believe that *Rango Cards* will provide a comprehensive experience on the topic, improving the students’ autonomy, motivation, and pleasure of learning.

**Trial registration:**

RBR-72zvxv June 29, 2018; Retrospectively registered.

## Background

Obesity is a serious public health problem that affects different age groups worldwide. Surveys on health and nutritional status of Brazilian students have shown an exponential growth in the prevalence of overweight and obesity in recent decades [1–3]. This situation has important health implications [4] and results from multiple factors, including modifiable conditions such as an inadequate diet. As the literature clearly demonstrates, the high consumption of ultra-processed foods correlates with the prevalence of overweight and obesity among adolescents and adults [5–10].

The dietary practices of Brazilian adolescents are frequently considered inappropriate because they include regular intake of soft drinks, artificial juices, sweets, sugar, and fried and high-fat foods; low intake of fruits and vegetables; and irregular eating habits, as meals are replaced with snacks and breakfast is missed [1–3, 11–13]. The Brazilian Study of Cardiovascular Risks in Adolescents (ERICA), with 71,791 participants, reported that the consumption of saturated fats, sugar and sodium was higher than the recommended upper limits; conversely, the recommended consumption of calcium and vitamins A and E was not reached [14].

Coping with that public health issue is a major challenge because eating behavior is influenced by the dietary environment and by social, cultural, and infrastructure conditions [15]. Current World Health Organization (WHO) guidelines indicate that a set of regulatory provisions, educational actions, and breastfeeding and healthy diet measures should be adopted to prevent obesity in children and adolescents [16].

Dietary guidelines are food and nutrition education (FNE) tools aimed at promoting healthy lifestyles and preventing diet-related diseases at the individual and collective level [17]. In Brazil, the *Dietary Guidelines for the Brazilian Population* provide a framework for educational actions, and their implementation should be based on the use of methods that encourage people to discuss and understand the concepts involved in eating behavior [18, 19]. To disseminate their content, some aspects should be considered, including the social market and the available technological innovations [20], such as the Internet and mobile devices (tablets and smartphones) [21].

Mobile health (mHealth) integrates mobile technology with public health practices with the purpose of improving communication and achieving healthy lifestyles [22]. Studies have reported that mHealth interventions may increase access to health care services; initiate the process of behavior changes or increase motivation for small changes; and spread knowledge, especially if aimed at receptive consumers [23–26]. Adolescents and young adults form the audience that readily accepts and adopts new technologies; thus, the diffusion effect may be stronger among them [27].

When assessing behavior changes in the field of food and nutrition, another important term is mobile nutrition, or mNutrition. This type of technology is based on repeated contact and exposure to messages that increase knowledge and raise awareness about nutrition, being a useful tool to change attitudes and support healthier lifestyles in terms of dietary habits [28, 29].

Currently, there is consistent evidence of the benefits of using mobile tools to improve health behaviors, especially when they are developed within a theoretical framework [30]. According to Sparapani [31], social cognitive theory [32] can be applied in health promotion to link interactive technologies and behavior changes. However, studies assessing the effectiveness of such interventions are still scarce [29, 33], requiring further research [34, 35]. Recently, Melo et al. [30] systematically reviewed 11 studies on the use of technologies in nutritional interventions for adolescents. The authors suggest that long-term interventions tend to be more successful when they involve frequent exposure to technological resources and a theoretical component aimed at a single health behavior change. The most commonly used theoretical framework in the reviewed studies was social cognitive theory.

mHealth strategies include the use of mobile applications (apps) as a resource for health improvement [36]. These mHealth apps have been increasingly downloaded [37], including digital games [38, 39]. In their review, Melo et al. [30] indicated that games seem to be promising tools for nutritional interventions, as all reviewed studies using this type of technology were effective, especially among young people.

Games are interactive systems that allow a more flexible relationship with the content, thus the player takes the leading role in the learning process. Their potential contribution depends on the player’s comprehension of the concept of the game and on the process of game design and its consequences [40, 41]. Schell [40] says that interactivity is a key element to understand the game. Without its interactive nature, the game becomes a linear narrative, similar to videos or books [42].

To be an engaging learning experience, the game must have the following attributes: gameplay, rules, goals, interactions, adaptations, actions, feedback, winning, conflicts, resolutions, and interpretations, all of them working interconnected [39]. Games designed to improve people’s cognitive and intellectual skills [43] are called educational games, learning games, or serious games [38, 44]. Several benefits have been attributed to game-based learning [43, 45, 46], including changing people’s behavior [44, 47], which justifies its increasing importance in the game industry [48].

To actually play the game, the player must be driven by an intrinsic motivation, i.e., the sense of pleasure generated by the interaction [49]. The fascinating feature of the game is responsible for conducting the player’s experience in the universe of the game. The boundary between the game world and the real world is called the magic circle [50]. Because the act of staying in the magic circle is voluntary, the game must be designed to provide a comprehensive experience [51], in a process known as game design.

Game design is the art of organizing and aligning the different elements that form a game, which are known as the elemental tetrad and include story, aesthetics, mechanics, and technology [40]. With respect to the game mechanics, the process of learning how to play the game should provide the intended knowledge and skills [52]. The player’s immersion is based on the process of regulation of the difficulty level presented by the game [40, 42, 53]; thus, hard games may cause frustration while very easy games may be tedious [54]. Regarding the story, there is a strong need to plan a transversal progression throughout the game [55], which allows a fluid immersion of the player in the magic circle.

Several FNE actions have been described in the literature; however, the challenge of adapting food and nutrition guidelines to achieve effective changes in health behavior remains [12], especially when involving the adoption of innovative methods of health education [56]. Digital games have the potential to support the learning process, providing greater personalization, dynamism, and autonomy. They are considered promising tools to promote changes in dietary behavior, which is the scope of this study.

Therefore, this study aims to outline a nutritional intervention for school-aged adolescents from the Federal District, Brazil, whose object is a digital game aimed at promoting healthy dietary practices. It is randomized study with intervention and control groups.

## Methods

### Study design

This protocol describes the development of a game-based nutritional intervention to implement the *Dietary Guidelines for the Brazilian Population* [19] among Federal District students. A randomized study with intervention and control groups will be conducted. The study was approved by the Research Ethics Committee of the University of Brasília School of Health Sciences (protocol no. 53175015.6.00000030) and funded by the Research Support Foundation of the Federal District, Brazil (FAPDF). The school units, the students, and their parents or legal guardians will provide written consent before the beginning of the study. We intend to visit each school unit at least three times during the study.

### Participants

#### Eligibility criteria

The studied population will consist of 1st-year high school students from private Federal District schools [57]. We chose private schools because the intervention involves a digital game that will be installed in student-owned mobile devices (smartphones and tablets), which tend to be expensive and thus more accessible to private school students than public school students. This study has no exclusion criterion.

#### Sample size and recruitment

The sample size was calculated considering the prediction of a 1.5 point increase in the food knowledge score after the intervention, with a standard deviation of 2.9 [58], a significance level of 5%, and power of 80%. Under these conditions, the minimum sample should be of 60 individuals per group, with control of the participants’ gender. Adding an estimated 40% loss between the pre- and post-intervention moments, the minimum total sample is defined as 168 individuals (84 per group). Each class in private schools has approximately 35 students [57]. Considering an estimate enrollment of 21 participants per class (40% of refusal), we expect to enroll 8 schools in the study, which will be randomly selected to form the study groups: 4 in the intervention group and 4 in the control group.

**Fig. 1:**
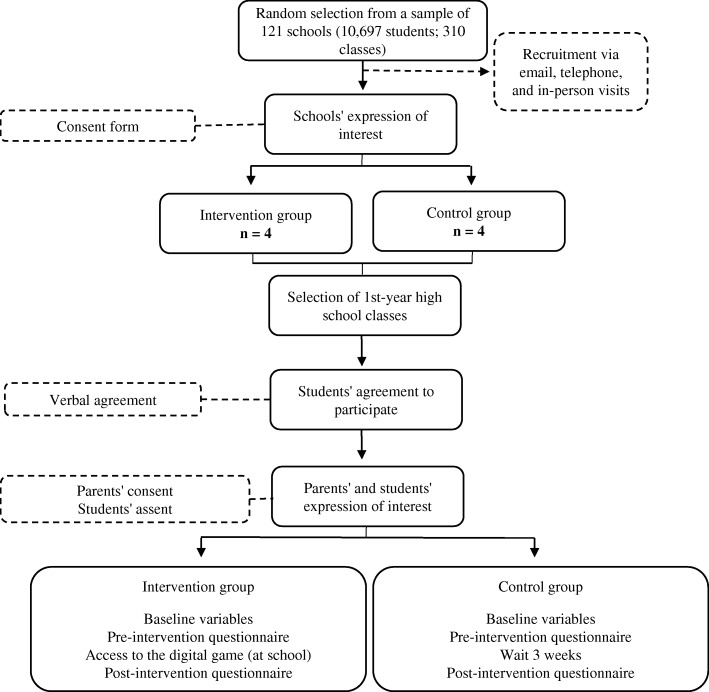
Study Procedure Flow Diagram

To recruit the selected school units, we will use email, telephone, and in-person visits to contact each school and explain our research. After the schools agree to participate and sign a consent form, we will select 1st-year high school classes and schedule visits to explain the study to students. Those who verbally agree to participate will be asked to request the written consent of their parents or legal guardians. Finally, the students’ assent will be obtained. Figure 1 shows the flowchart of recruitment and intervention of the study.

### Intervention

#### Rango cards development process

A team of dietitians, Fira Soft® game developers, and adolescents was involved in the development of a digital game. As a preliminary activity, the hired company informally collected opinions about two game mechanics from adolescents and young adults (13–24 years) who play games frequently. We chose the mechanics and format of Hearthstone® (Blizzard Entertainment), named the best multiplayer game by Google Play in 2017 [59]. After setting the basic structure of the game, the concepts of art and user interface were created, as well as the game design document. A convenience sample of adolescent players was selected to be part of the production team and participate in all assessments of viability, usability, and attractiveness of the mobile application. They were all interested in the topic and their age group (14–16 years) was compatible with the target audience of the game.

The developed tool can be classified as a serious game. It is free, compatible with Android and iOS devices, and available from Google Play and App Store since April 2017. The name *Rango Cards* was chosen because it links the word “rango” (an informal word in Brazilian Portuguese to refer to food) to the word “cards” (the type of game). The mechanics of card games explores the strategic potential of each player. Social cognitive theory was used to develop the game [32, 60].

The visual style of the game was based on the preferences expressed by the adolescents, favoring a cheerful, simple, and friendly atmosphere. The cards were differentiated by color, in line with the logic of other games and also the traffic lights. Thus, green cards represent healthy foods and/or meals that should be consumed regularly; yellow cards represent foods that should be consumed moderately; and red cards represent foods that should be avoided, as recommended by the *Dietary Guidelines for the Brazilian Population* [19]. There are also cards of characters and healthy practices, such as cooking and having meals at clean environments and in the company of other people, thus showing that the concept of a healthy diet is not associated only with food intake.

The game uses a real-time system of sodium, sugar, and fat content meters, which change when the player uses cards of processed or ultra-processed foods. In the game, these are called industrialized and mega-industrialized foods, respectively. The meters influence the playability and the fate of the player in a match. At first, the effect seems mild, but as the player uses more unhealthy food cards the damages become more evident, impacting health and energy levels and the remaining time to play.

The virtual environment where the narrative of *Rango Cards* takes place is a school with typical spaces (schoolyard, library, auditorium) and characters, except for the third phase, when the player visits a restaurant. The scenario for the matches is a dining table. Characters are ethnically diverse and wear simple clothes to facilitate the player’s identification with the illustrations. Features of Japanese animation (anime) and American comic books were used. To engage players, contextualize the game, and simplify the transposition of concepts, the phases were interspersed with dialogue scenes, which tell a linear narrative and involve all the game characters. This contributes to increase the immersion in the game experience and the intrinsic motivation to play. As recommended by Hingle et al. [61], dialogues are short and objective.

The difficulty level of the game oscillates between easy and hard. This required a two-step calibration, as follows: first, the cards were balanced and then the decks were compiled, one by one, in the order they appear in the game. Regarding card attributes, the greater the effect of a card in the game, the higher its cost to the player, i.e., the higher the amount of energy required to make a move. The idea is to show that choices are significant and may result in winning or losing. In the first phase, the deck consists of 23 cards; from the fifth phase on, the player has access to the full 44-card deck. The logic is to gradually introduce new concepts and improve the player’s skills.

#### Game components

**Fig. 2:**
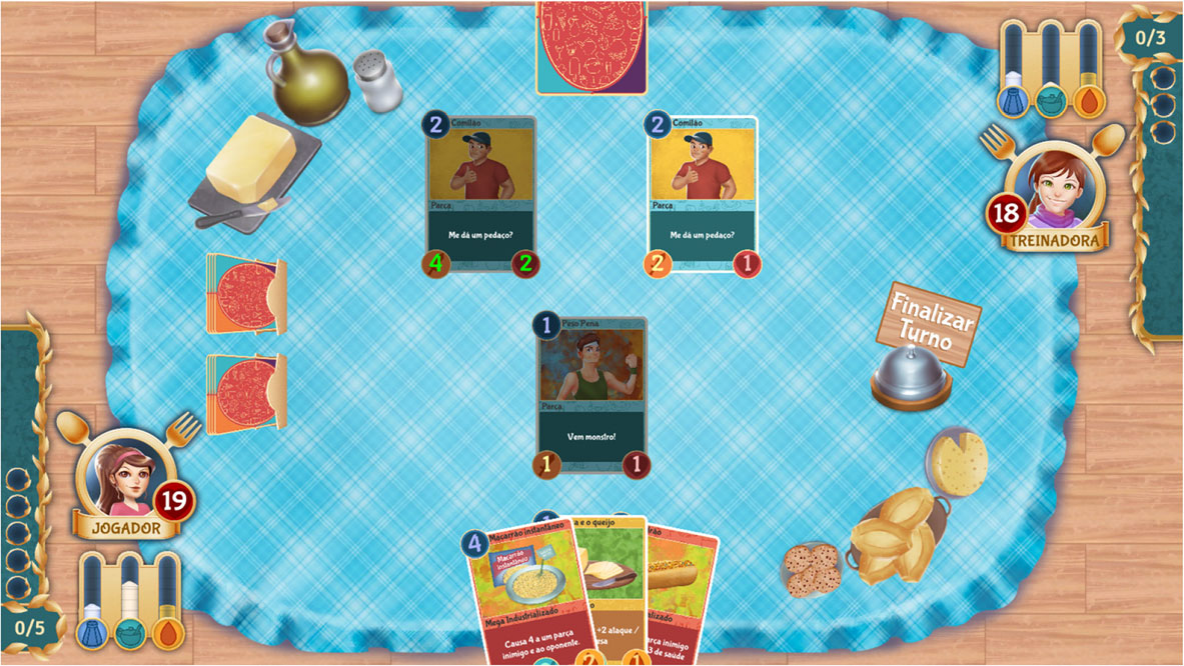
Screenshot of Rango Cards’ interface

To initiate the game, the player needs to complete a brief profile and choose a female or male avatar. Then, a school character invites the player to participate in a new health and diet training by playing a card game. Each phase includes computer-controlled opponents, i.e., this is not a player vs. player game. The game environment components are the following: a) energy markers; b) card deck; c) avatar, opponent, and foods, which are grouped according to the classification described in the *Dietary Guidelines for the Brazilian Population* [19]. Each character has a health score accompanied by sodium, sugar, and fat content meters. Figure 2 shows the game components of *Rango Cards*.

**Fig. 3:**
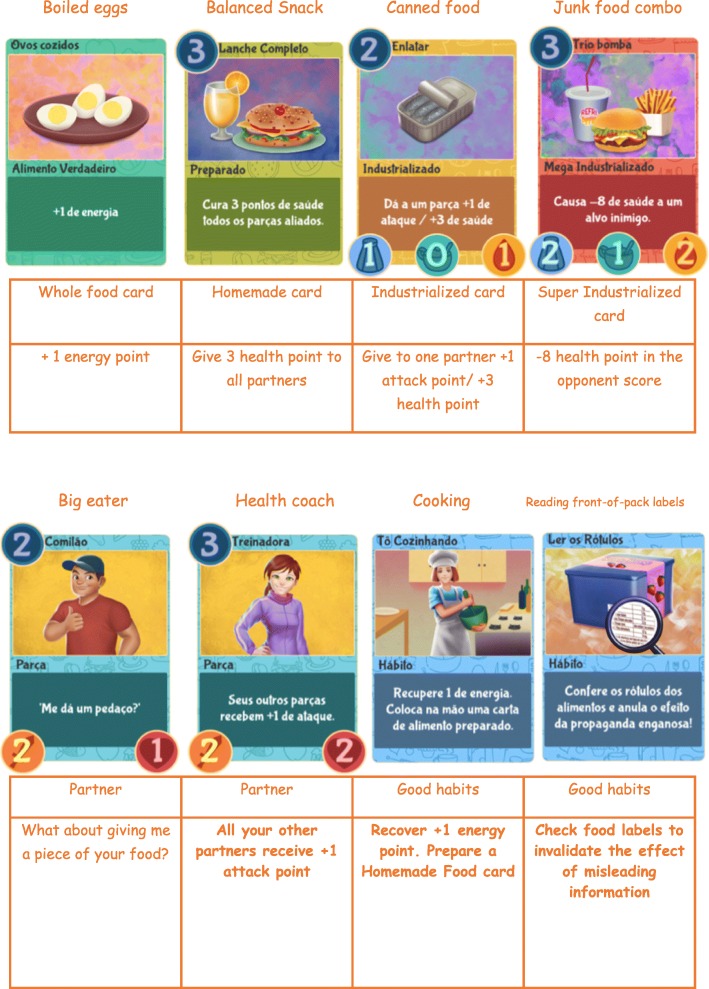
Example of cards used in the game

The playability of *Rango Cards* is supported by the mechanics of energy. The player has a fixed amount of energy at the beginning of each match and must use it to play the cards. It is worth mentioning that there is no connection between the words “energy” and “calories.” In the first phase, a dynamic tutorial explains the function of energy in the matches. This system is fed back by the player’s choices; thus, the use of healthy food/meal cards is a positive reinforcement and provides rewards (energy) to influence automatic behavior [62]. The cards for foods/meals, dietary practices, and characters are named and subdivided as follows:

- ***Real food***: green cards representing *in natura* (fruits, water, etc.) and minimally processed foods (natural fruit juice, boiled eggs, etc.). They increase the upper energy limit available to the player at the beginning of each turn, i.e., they are crucial to the game.
- ***Prepared meal***: Green cards representing meals cooked on the spot. They are introduced in the third phase, in a restaurant where family recipes passed on from generation to generation are prepared. These cards provide health points, greater attack power, or rewards in the form of new cards, thereby showing the benefits of making healthy choices, especially when eating out.
- ***Industrialized food***: yellow cards representing processed foods (cheese, canned sardine, etc.), which underwent several processes in the food industry and are considered modified versions of the original food [19]. These cards affect sodium, sugar, and/or fat content meters; conversely, they usually have positive effects on the game characters, reinforcing the *Dietary Guidelines* [19] with respect to the need to restrict or consume small portions of these foods.
- ***Mega-industrialized food***: red cards representing foods and meals formulated with an excessive amount of ingredients, which characterizes ultra-processed products. They usually provide the player with some skill to directly harm the opponent or the rival characters, reinforcing the idea that these foods and meals cause health damages. They also affect sodium, sugar, and/or fat content meters.
- ***Characters***: named “Buds” (game buddies), they help the player during the matches by attacking and harming the opponent and the rival Buds or defending the player.
- ***Habit***: cards related to the act of eating and its dimensions, including the importance of choosing appropriate places to buy *in natura* foods; the importance of breakfast; the recommendation of home cooking and eating meals at regular times and clean spaces; the time and attention given to the act of eating; the importance of eating in the company of other people; and the need to critically analyze food advertising and read product labels. These elements reflect the concept of commensality described in the *Dietary Guidelines for the Brazilian Population* [19]. During a match, habits provide the player with benefits such as additional health points, energy or time to play; addition and/or replacement of cards; reversal of negative effects of other cards; and restoration of Buds’ attack power. Figure 3 shows the cards used in *Rango Cards*.

The goal of the game is to make the opponent lose health points, which depends on at least two factors. The first is luck, considering that cards are dealt randomly and there is no predetermination of winning or losing. An exception is the third to last phase, when a new concept is explained and a new card is introduced to the game. The possibility of making choices that lead to different endings and having performance indicators provides autonomy and increases skills and intrinsic motivation [62]. The second factor is the adopted strategy. The player should use the cards logically and spend the energy provided by the cards that allow a longer match time, thus saving as many health points as possible.

*Rango Cards* is divided into seven phases, addressing the following themes: food classification; healthy practices; importance of home cooking; food marketing focused on misleading advertisement; and food label reading. Theme selection and phase order were designed to provide a learning experience. The players are repeatedly exposed to some concepts, as Zajonc recommends [63], encouraging positive food-related attitudes.

The hypothesis is that the game may increase knowledge regarding an adequate and healthy diet as well as self-efficacy in the adoption of healthy practices, which are considered primary outcomes. Secondary outcomes are positive changes in dietary consumption, perceptions, and practices.

#### Preliminary evaluations

To evaluate the game before it was available from application stores, a convenience focus group with seven 1st-year high school students from a Brazilian private school was selected. In this group, impressions about the game and its potential as an educational tool were collected, including the following: *“As you play the game, you learn to develop strategies, eat healthy foods and balanced meals, and sometimes you can use industrialized foods. If you stop to think about it [...], this method is very cool”*; and *“If it was an explanatory thing I wouldn’t play it [...], the cool thing is to understand that if your sugar level is high your health gets worse.”* Qualitative evaluations revealed that the audience was involved, engaged, and interested in the tool, which is essential to the main goal of the game. Adaptations in the mechanics and error adjustments were performed with the purpose of increasing the likelihood of a successful game.

Game management reports from March 2018 informed that *Rango Cards* has been downloaded more than 4050 times after 11 months available from application stores; average rating is 4.9 stars; the most commonly used store is App Store (91%), and the most common device is iPhone (94%). Another positive indicator is that *Rango Cards* was awarded the Best Serious Game in the SBGames 2017, the greatest Latin American academic event in the field of games and digital entertainment.

#### Intervention group

Adolescents in the intervention group will be instructed to play the game, and the first contact will occur at the school. Because of its autonomous nature, the intervention will not be included in the school curriculum. To increase the intervention group’s motivation, an environment of challenge and social interaction will be created among the students of each school, based on a competition of time spent to finish the game. These aspects are considered important by adolescents who play games [33, 62, 64]. The students will be able to play *Rango Cards* at school and also at the time and place which are more convenient for them. The period to play will be 3 weeks, and then a post-intervention questionnaire will be applied. This autonomy, which reflects “the will to perform a task,” aims to improve intrinsic motivation [64].

#### Control group

Adolescents in the control group will not be instructed to play the game nor will receive any healthy diet material, i.e., the researchers will not provide them with any additional material during the study.

#### Measures

Both groups will complete research questionnaires before and after the nutritional intervention. The following variables will be investigated: age, sex, family income, maternal level of education, dietary perceptions and practices, food knowledge, and self-efficacy in the adoption of healthy practices. Family income and maternal level of education will be evaluated using questions from population surveys performed in Brazil periodically [65]. Dietary perceptions will be based on a scoring system in which participants will evaluate how healthy their diet is and how much they know about the topic. Dietary practices will be assessed using the weekly frequency of consuming healthy (beans, vegetables, and fruits) and unhealthy foods (ultra-processed foods, fried snacks, soft drinks, sweets, and fast food), the frequency of having breakfast, lunch, or dinner with parents or legal guardians, and the frequency of eating while watching TV or studying [3]. Food knowledge and self-efficacy in the adoption of healthy practices will be based on the content of the game and on items from previous studies with adolescents and young adults [13, 66, 67]. The participants will be asked to analyze some statements and express their level of agreement using a 5-point Likert-type scale.

The questionnaires will be completed and supervised by at least two researchers. Pre- and post-intervention questionnaires will comprise the same questions, except for the sociodemographic variables, which will be investigated only at the first moment. The instruments will be previously tested in a convenience sample of adolescents who will not be included in the main study. This activity will assess clarity and comprehension of each question, providing an analysis of structural items such as language, progressive logic, question order, and writing strengths and/or weaknesses. The necessary adjustments will be performed before the beginning of the main study.

#### Data analysis

After data collection, the questionnaires will be entered into electronic spreadsheets. To ensure data reliability, data will be double entered. Sociodemographic variables will be analyzed descriptively and expressed as mean, standard deviation, and frequencies. The Pearson’s chi-squared test will be used for sex and age, while the Mann-Whitney U test will be used for family income and maternal level of education. To assess the impact of the intervention, unpaired analyses will be performed using the Student’s t-test for continuous numerical variables (dietary perceptions) and Likert-type scales (food knowledge and self-efficacy). The Mann-Whitney U test will be used for dietary practices and food consumption. The significance level will be set at 5%, and the statistical analyses will be performed using the Statistical Package for the Social Sciences (SPSS), version 21.0.

## Discussion

This randomized study will assess the impact of a nutritional intervention on school-aged adolescents from the Federal District, Brazil. The object is a digital game that was developed specifically for this study. As the literature suggests, mHealth interventions result in high levels of acceptance and improvement of health behaviors [25, 29, 33]. The study proposal innovates by introducing the concept of an adequate and healthy diet described in the *Dietary Guidelines for the Brazilian Population* [19]. In addition, the game mechanics was inspired in a major worldwide success. According to Bakkes, Tan, & Pisan [68], a player-centered game design leads to a stronger connection between the player and the technology, ensuring increased playability and satisfaction.

Although this study innovates in terms of design and aims, it also has some limitations, especially because social or environmental factors may interfere with the acceptance of the game [29]. Therefore, in the process of developing *Rango Cards*, a comprehensive experience was designed considering target audience characteristics, autonomy, motivation, and pleasure of learning, which may increase the effectiveness of the intervention in establishing sustainable behavior changes [62, 69, 70].

This intervention will contribute to the development of specific tools for FNE strategies focused on adolescents, considering that there is still a shortage of educational material for this audience. Thus, this study is expected to become a theoretical and methodological benchmark for other educational initiatives based on the use of digital tools to promote an adequate and healthy diet.
